# The Pharmacological Frontier in Pediatric Heart Failure Management: Innovations and Prospects

**DOI:** 10.7759/cureus.51913

**Published:** 2024-01-08

**Authors:** Sohilkhan R Pathan, Vishal V Bhende, Kruti B Sharma, Vishal A Patel, Dinesh M Gangoda, Tanishq S Sharma

**Affiliations:** 1 Clinical Research Services (CRS), Bhanubhai and Madhuben Patel Cardiac Centre, Shree Krishna Hospital, Bhaikaka University, Karamsad, IND; 2 Pediatric Cardiac Surgery, Bhanubhai and Madhuben Patel Cardiac Centre, Shree Krishna Hospital, Bhaikaka University, Karamsad, IND; 3 Community Medicine, Sal Institute of Medical Sciences, Ahmedabad, IND

**Keywords:** multidisciplinary approaches, combination therapies, precision medicine, pharmacological interventions, pediatric heart failure

## Abstract

Pediatric heart failure, encompassing a diverse range of conditions, imposes a significant burden despite its relatively low incidence. The contemporary landscape, with infants constituting a majority of admissions, underscores the need for specialized attention. This editorial delves into the evolving pharmacological interventions for pediatric heart failure, emphasizing the nuances of managing congenital heart defects, genetic factors, and diverse etiologies. The goal is to contribute knowledge that addresses the unique needs of children and explores innovations promising to redefine care standards. The narrative navigates through the current state of pediatric heart failure management, unique considerations, emerging pharmacological innovations, precision medicine, addressing underlying causes, combination therapies, clinical trials, and ethical considerations. Each section contributes to a comprehensive understanding of the evolving landscape and sets the stage for potential future directions in pediatric heart failure care.

## Editorial

Pediatric heart failure represents an intricate clinical and pathophysiologic syndrome encompassing a diverse cohort of patients dealing with conditions such as congenital heart disease (CHD), cardiomyopathy, infectious and inflammatory diseases, oncologic processes, metabolic syndromes, renal failure, and malnutrition [1]. Despite the relatively low estimated incidence of heart failure, ranging from 0.9 to 7.4 per 100,000 children, it imposes a substantial burden of morbidity and mortality, with an in-hospital mortality rate ranging from 7% to 26% [2]. In the contemporary era, infants constitute the majority (64%) of heart failure admissions in patients aged 18 years or younger [3]. The predominant cardiac diagnosis at admission is CHD (69%), followed by arrhythmias (12-15%), cardiomyopathy (13-14%), and myocarditis (~2%). Recent analyses have brought to light that primary presenting complaints often involve respiratory and/or gastrointestinal symptoms, which may mimic more common pediatric illnesses, thereby leading to inaccurate and/or delayed diagnoses [4].

Pediatric heart failure poses unique challenges in cardiology, often originating from congenital defects or genetic factors, necessitating specialized attention for tailored treatment. Despite the effectiveness of current medications, the diverse causes and developmental differences demand innovative solutions. This editorial explores the evolving landscape of pharmacological interventions for pediatric heart failure, aiming to contribute to knowledge of children's unique needs. Emphasizing the need for a nuanced understanding, the paper navigates complexities and highlights promising innovations that could redefine care standards. The goal is a future where personalized interventions improve the quality of life for affected children beyond just symptom relief.

Pediatric heart failure management involves a dynamic interplay of medical advancements, clinical expertise, and a nuanced understanding of unique challenges. Rooted in congenital heart defects, genetic factors, or acquired conditions, comprehensive care is crucial. Early intervention relies on advanced diagnostics like echocardiography and cardiac MRI. Pharmacological treatment aims to alleviate symptoms, with medications like angiotensin-converting enzyme (ACE) inhibitors and beta-blockers, but variability in response highlights the need for personalized therapy. Surgical interventions, including correction of defects and heart transplantation, are pivotal when pharmacological approaches fall short. Multidisciplinary teams, comprising various healthcare professionals, ensure holistic patient care, addressing both cardiac and psychosocial needs. Ongoing research and clinical trials contribute to expanding treatment options, but challenges persist, such as donor scarcity and the long-term effects of interventions. Despite these, the evolving landscape emphasizes collaboration, research, and personalized care to meet the complex needs of pediatric heart failure patients.

Managing pediatric heart failure requires a subtle approach considering developmental stages, individualized care, and unique etiologies. Challenges arise from ongoing growth, impacting organ development in infants and toddlers. Tailoring treatments to age-specific needs is crucial, acknowledging physiological differences. Unlike adult heart failure, pediatric cases often result from congenital defects, genetic factors, or childhood-specific conditions. Understanding and addressing these causes are essential for targeted interventions and optimal outcomes. Pediatric heart failure extends beyond physiology to impact psychosocial and developmental well-being. A holistic approach integrates psychosocial support, educational resources, and developmental assessments. Family involvement is integral, recognizing parents and caregivers as crucial in day-to-day management and emotional coping. Transitioning to adult care in adolescence is critical, requiring continuity, addressing changing medical needs, and facilitating a smooth transfer to adult services. Overall, pediatric heart failure management demands a comprehensive understanding, and subsequent sections will explore the evolving pharmacological landscape and innovations for the future.

Exploring the frontier of emerging pharmacological innovations in pediatric heart failure management reveals promising strides. Recent advancements focus on novel medications tailored to pediatric populations, aiming to enhance cardiac function and optimize treatment efficacy. Precision medicine guides the development, tailoring interventions based on genetic and biomarker profiles for more effective therapies. Combination therapies have gained prominence, offering a multifaceted approach to managing heart failure with improved efficacy and minimized adverse effects. Innovative drug delivery methods, including liquid formulations and age-appropriate dosage forms, address unique challenges in pediatric patients. Ongoing clinical trials contribute to the expanding arsenal of treatment options, with a focus on safety, efficacy, and long-term effects. Despite the promise, ethical considerations play a crucial role in balancing the imperative to advance treatments with rigorous testing and patient safety. In summary, the evolving landscape of pharmacological interventions for pediatric heart failure is marked by precision medicine, combination therapies, and innovative drug delivery methods. Subsequent sections will explore the impact of these innovations on outcomes and the potential trajectory of pediatric heart failure pharmacological interventions.

In pediatric heart failure management, precision medicine and personalized pharmacotherapy have emerged as transformative approaches. Tailoring medical interventions to the unique characteristics of each child, including genetic, biomarker, and patient-specific factors, marks a paradigm shift. Genetic testing identifies hereditary factors, guiding targeted interventions and predicting treatment responses. Biomarkers offer real-time insights, aiding in early detection and personalized treatment adjustments. This individualized approach extends beyond a one-size-fits-all model, recognizing the diverse genetic landscape and varying responses across different age groups. Implementing personalized pharmacotherapy requires collaboration among healthcare providers, researchers, and families, with a focus on education and support. While precision medicine holds promise, challenges such as access to genetic testing and ethical considerations persist. Balancing personalized approaches with equitable access remains crucial. In conclusion, precision medicine represents a paradigm shift, holding the potential to improve outcomes in pediatric heart failure. Subsequent sections will explore ongoing innovations and prospective directions in the pharmacological landscape for pediatric heart failure.

Addressing the root causes of pediatric heart failure is crucial in contemporary cardiac care for children. Unlike adult cases, pediatric heart failure often stems from congenital defects, genetic factors, or childhood-specific conditions. This complexity underscores the need for tailored interventions targeting diverse etiologies. Congenital heart defects, prevalent contributors, require surgical correction with advances improving long-term outcomes. Genetic factors necessitate testing for mutations and personalized treatment plans, with genetic counseling guiding family decisions. Acquired conditions like myocarditis involve multifaceted approaches, including pharmacological interventions and, at times, transplantation. Metabolic factors, such as diabetes and inflammatory conditions like Kawasaki disease, impact pediatric heart failure, requiring comprehensive management. Collaborative efforts between specialists are essential, and ongoing research explores underlying causes, offering promising avenues for targeted interventions. Continued focus on understanding and addressing root causes remains pivotal for optimizing outcomes. Advances in surgical techniques, genetic diagnostics, and targeted interventions align with the goal of improving the lives of children facing heart failure. Subsequent sections will explore innovative pharmacological strategies and their promise for the future of pediatric heart failure care.

**Figure 1:**
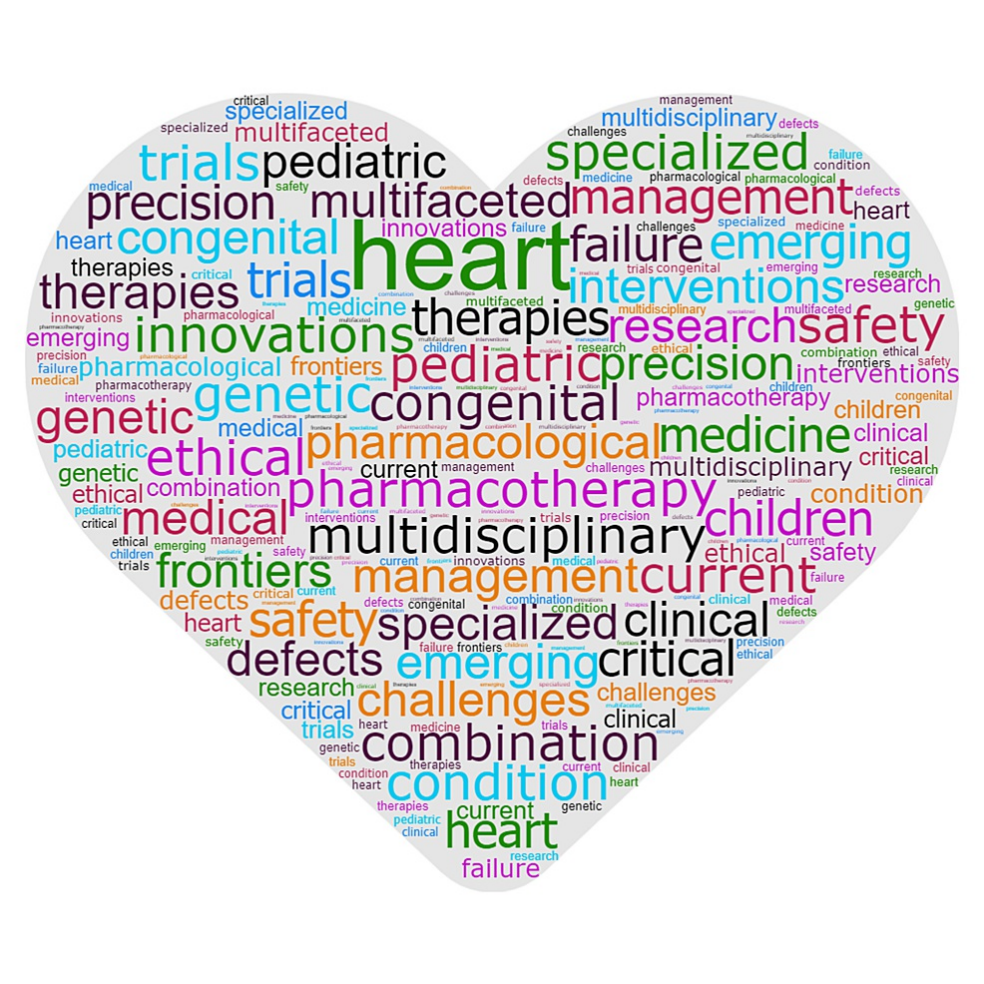
Word cloud of pharmacological considerations in the management of pediatric heart failure

The evolving landscape of pediatric heart failure management embraces innovative strategies, shifting towards combination therapies and multidisciplinary approaches. Combination therapies involve using multiple drug classes with complementary mechanisms of action, addressing diverse etiologies, and aiming for a synergistic impact on cardiac function in children. Figure 1 depicts a word cloud of pharmacological considerations in the management of pediatric heart failure.

Conclusion

In conclusion, pediatric heart failure management is shaped by dynamic interplays of evolving pharmacological strategies, multidisciplinary approaches, and ethical considerations. The complexities demand nuanced understanding, individualized care, and addressing unique etiologies. Innovations in precision medicine, combination therapies, and personalized approaches offer promise for optimizing outcomes. Addressing underlying causes, refining treatment modalities, and incorporating insights from clinical trials guide progress. Ethical imperatives underscore responsibility, emphasizing transparent communication and a delicate balance between innovation and patient safety. Ongoing research initiatives deepen our understanding, while collaborative efforts drive a collective commitment to advancing care standards. Navigating the future requires attunement to evolving landscapes and embracing principles of innovation, ethics, and multidisciplinary care. This collective endeavor aims to enhance the quality of life for children facing the challenges of heart failure. The journey toward optimal pediatric heart failure care reflects a commitment to innovation, compassion, and the well-being of our young patients.
